# Stereotactic ablative radiotherapy of 60 Gy in eight fractions is safe for ultracentral non‐small cell lung cancer

**DOI:** 10.1111/1759-7714.13335

**Published:** 2020-02-03

**Authors:** Dan Yang, Jianing Cui, Jun Zhao, Jing You, Rong Yu, Huiming Yu, Leilei Jiang, Dongming Li, Bo Xu, Anhui Shi

**Affiliations:** ^1^ Department of Radiation Oncology, Key Laboratory of Carcinogenesis and Translational Research (Ministry of Education/Beijing) Peking University Cancer Hospital & Institute Beijing China; ^2^ Department of Radiation Oncology, Beijing Tsinghua Changgung Hospital School of Clinical Medicine Beijing China; ^3^ Department of Thoracic Oncology I, Key Laboratory of Carcinogenesis and Translational Research (Ministry of Education/Beijing) Peking University Cancer Hospital & Institute Beijing China

**Keywords:** Clinical outcomes, dosimetry, SABR, ultracentral NSCLC

## Abstract

**Background:**

There is no consensus on the definition or recommended radiotherapy treatment of ultracentral non‐small cell lung cancer (NSCLC). Here, we report our institution's experience in treating ultracentral lung cancer patients with stereotactic ablative radiotherapy (SABR) of 60 Gy in eight fractions.

**Methods:**

We retrospectively reviewed the outcomes of 21 ultracentral NSCLC patients treated with 60 Gy SABR in eight fractions. We defined ultracentral lung cancer as the planning target volume (PTV) directly abutting or overlapping central structures, including the proximal bronchial tree, heart, and great vessels but not the esophagus. The Kaplan‐Meier method was used to estimate overall survival (OS), progression‐free survival (PFS) and local control (LC). Toxicity was scored per the CTCAE v4.03.

**Results:**

The median follow‐up time was 15 months, and the median OS was 15 months. The one‐ and two‐year OS rates were 87.5% and 76.6%, respectively. The one‐ and two‐year PFS rates were 71.1% and 64.0%, respectively. The one‐ and two‐year LC rates were 92.9% and 92.9%, respectively. The rate of grade 2 treatment‐related toxicities was 19.1%. There was no grade ≥ 3 treatment‐related toxicity.

**Conclusion:**

SABR of 60 Gy in eight fractions is feasible for ultracentral NSCLC.

## Introduction

Non‐small cell lung cancer (NSCLC) is the leading cause of death from cancer worldwide.^1^, ^2^ With the development of radiotherapy, stereotactic ablative radiotherapy (SABR) has become an effective treatment for NSCLC.^3^, ^4^, ^5^, ^6^ RTOG 0236 suggested that peripheral stage I non‐small cell tumors treated with SABR could achieve acceptable local control (LC) and overall survival (OS).^7^ A phase 3 randomized trial comparing SABR with surgery has suggested that SABR was better tolerated and might lead to better OS compared with surgery for operable clinical stage I NSCLC.^8^, ^9^ In the past, we preferred to treat peripheral lung tumors with SABR, which has been proven safe and efficient. However, we are currently trying to use SABR for patients with central lung cancer. Chang *et al*.^10^ defined a central/superior location as being within 2 cm of the bronchial tree, major vessels, esophagus, heart, trachea, pericardium, brachial plexus or vertebral body but 1 cm away from the spinal canal. RTOG 0813 stated that centrally located NSCLC is within or touching the zone of the proximal bronchial tree or adjacent to the mediastinal or pericardial pleura. Those patients were successively accrued onto a dose‐escalating, five‐fraction SABR schedule ranging from 10–12 Gy per fraction. The LC at two years was high, and grade ≥ 3 toxicity rates were acceptable.^11^

Recently, a new category of tumors named “ultracentral” has been proposed. Some reports have discussed the definition of ultracentral lung cancer. Tekatli *et al*.^12^ defined “ultracentral” lung cancer as centrally located NSCLC with planning target volume (PTV) overlapping the trachea or main bronchi. Chaudhuri *et al*.^13^ defined “ultracentral” as those with gross tumor volume (GTV) directly abutting the central airway. Raman *et al*.^14^defined it as PTV directly contacting or overlapping the proximal bronchial tree, trachea, esophagus, pulmonary vein or pulmonary artery.

At the same time, the toxicity associated with SABR for ultracentral tumors is also a main issue. Haasbeek *et al*.^15^ provided a regimen of 60 Gy in eight fractions to patients with central hilar tumors or tumors abutting the pericardium and mediastinal structures, with no excess toxicity. Chaudhuri *et al*.^13^ summarized ultracentral lung tumors treated with 50 Gy in 4–5 fractions, and patients with ultracentral tumors experienced no symptomatic toxicities over a median follow‐up time of 23.6 months. Based on those results, SABR seems safe for ultracentral tumors.

At present, there is no common view on the definition or the optimal radiotherapy dose for ultracentral lung cancer. Therefore, we define ultracentral lung cancer as the PTV directly abutting or overlapping central structures, including the proximal bronchial tree, heart, and pulmonary artery or pulmonary vein but not the esophagus. In this study, we report our institution's experience in treating ultracentral lung cancer patients with 60 Gy SABR in eight fractions. Here, we retrospectively examined the toxicities and outcomes of these patients.

## Methods

### Patients

After obtaining the local Institutional Review Board (IRB) approval, we conducted a retrospective review of all patients at our institution who were treated for ultracentral NSCLC using SABR at a dose of 60 Gy in eight fractions between April 2012 and March 2018. Ultracentral lung tumor was defined as the PTV directly abutting or overlapping central structures, including the proximal bronchial tree, heart, and great vessels but not the esophagus. We analyzed 21 consecutive patients with ultracentral lung cancer treated by SABR of 60 Gy in eight fractions. Patients with brain metastases or previous thoracic irradiation were excluded. Tumor stage was determined on the basis of chest computed tomography (CT), abdominal ultrasound/CT, brain magnetic resonance imaging (MRI), and bone scan or positron emission tomography (PET)‐CT by using the seventh edition of the tumor, node, and metastasis (TNM) system. All patients with stage I cancer must have had a PET‐CT scan for initial staging in order to determine the precise stage.

### SABR and dosimetry

All patients underwent enhanced chest CT and four‐dimensional (4D)‐CT localization scans. The scan thickness was 3–5 mm, ranging from the lower mandibular margin to the lower hepatic margin. Radiotherapy was performed with a Varian linear accelerator (True Beam or Edge) and 6–10 MV X‐ray. Internal gross tumor volume (IGTV) was delineated using a maximal intensity projection created by combining data from multiple 4D‐CT datasets at different breath phases and then modifying these contours by visual verification of the coverage in each phase of the 4D‐CT dataset. A 5 mm setup uncertainty margin was added to determine the PTV. We required that 95% of the PTV received a 100% prescription dose. All treatments were planned and delivered using volumetric modulated arc therapy (VMAT) technique. Acuros XB algorithm was used for dose calculation. Daily CT verification was conducted during each fraction of radiotherapy, and coverage of target volume and sparing of critical structures were verified.

### Follow‐up and assessment of outcomes

Follow‐up was conducted every three months for the first two years after radiotherapy and every six months for the next three years, followed by an annual follow‐up. The follow‐ups included physical examination, routine blood analysis, blood biochemistry, tumor markers, and imaging examinations (including chest CT, abdominal ultrasound/CT, brain MRI, and bone scan). In the first five years, bone scans were performed every six months. Tumor recurrence was confirmed by biopsy or dynamic observation of PET‐CT scan and tumor evaluation was based on the Response Evaluation Criteria in Solid Tumors (RECIST 1.1). Treatment‐related toxicities were evaluated according to the National Cancer Institute Common Terminology Criteria for Adverse Events (NCI CTCAE) version 4.03. We report on the maximum point doses (D_max_ in Gy), minimum doses received by small organs at risk (OAR) volumes (D_xcc_ in Gy), and volumes of OAR receiving xGy or more (VxGy in percentages).

### Statistical analysis

Statistical analysis was performed using IBM SPSS for Windows, version 22.0 (IBM, Armonk, NY). The time‐to‐event outcomes were calculated with the Kaplan‐Meier method. The date of first radiotherapy was defined as day 0 of follow‐up.

## Results

### Patients and tumor characteristics

**Figure 1:**
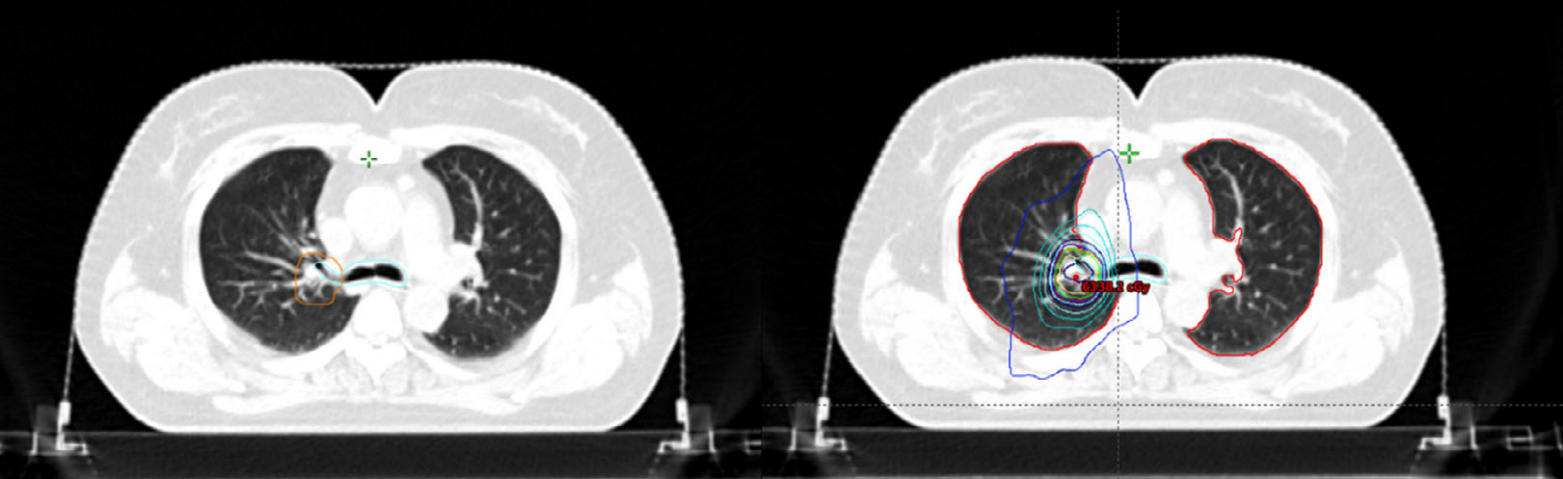
An example of ultracentral lung cancer (planning target volume in orange and proximal bronchial tree in blue) and its dose distributions.

A total of 21 patients with ultracentral lung tumors were included in this study (Table 1). Figure 1 shows an example of treated ultracentral tumors. The median age was 66 years (range 52–81 years). There were five patients in stage IV, three of whom had progression of primary lung cancer after chemotherapy or targeted therapy, the other two patients had oligometastatic lung cancer. Thus, we treated the stage IV patients with SBRT. Table 1 also shows that there were seven patients who received prior chemotherapy, two of which were in stage IV and needed chemotherapy. The rest of the patients received neoadjuvant chemotherapy but could not or refused to have surgery at last. Four patients received prior targeted therapy because they were in stage IV. Six patients had prior surgery, four of whom were treated with SBRT for a post‐surgery recurrence, the other two patients had second primary lung cancer after surgery. The median follow‐up time was 15 months (range 4–70). All tumors were treated with radiotherapy of 60 Gy in eight fractions, except for two patients with tumors who received a compromised PTV dose because their PTV overlapped a sinoatrial node or brachial plexus nerve.

**Table 1:** Patient and tumor characteristics

Characteristic	No. of patients (%) or median value (range)
Age, year	66 (52–81)
Gender	
Male	12 (57.14%)
Female	9 (42.86%)
Pathological diagnosis	
Squamous cell carcinoma	8 (38.10%)
Adenosquamous carcinoma	13 (61.90%)
PTV location	
Proximal bronchial tree	14 (66.67%)
Great vessels (pulmonary artery or vein)	9 (42.86%)
Heart	1 (4.76)
PTV size, cm^3^	36.5 (16.4–133.1)
T stage	
1a	1 (4.76%)
1b	5 (23.81%)
2a	6 (28.57%)
2b	1 (4.76%)
3	2 (9.52%)
4	6 (28.57%)
Disease stage	
I	9 (42.86%)
II	2 (9.52%)
III	5 (23.81%)
IV	5 (23.81%)
Prior treatment	
Chemotherapy	7 (33.33%)
Surgery	6 (28.57%)
Targeted therapy	4 (19.05%)

### Outcomes

**Figure 2:**
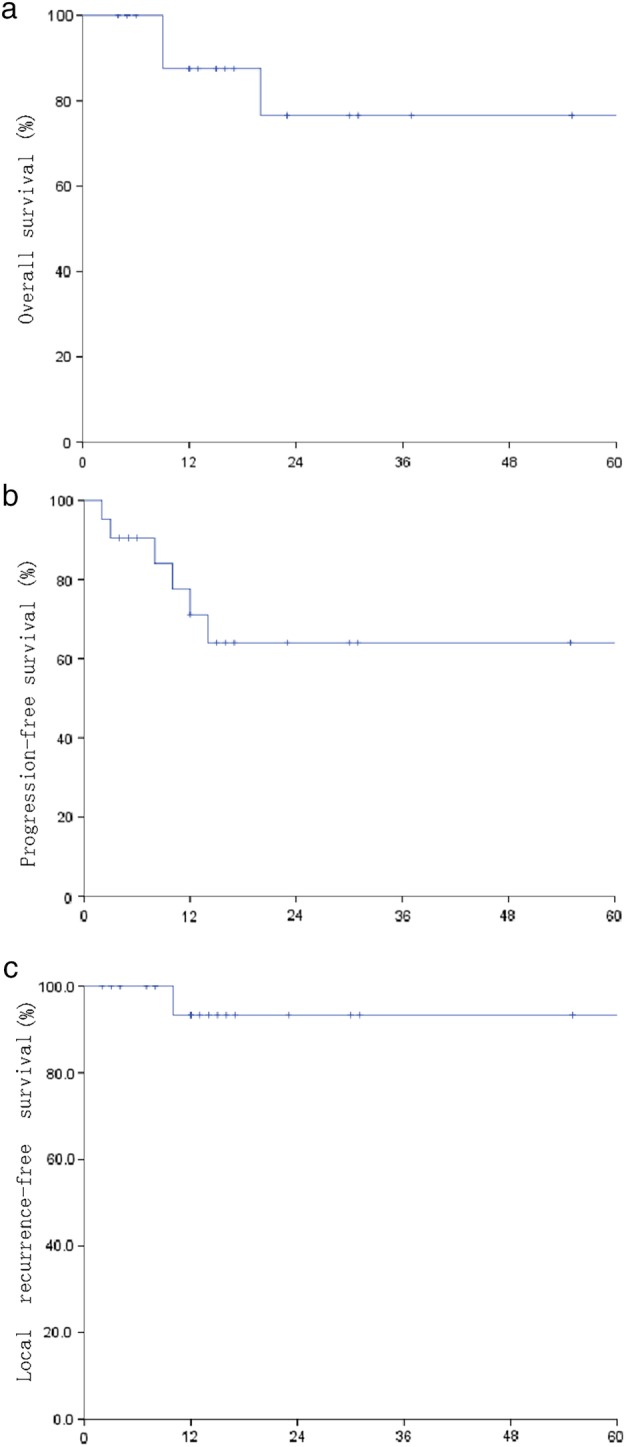
The overall survival (OS), progression‐free survival (PFS) and local control (LC) of all patients.

Among all patients, the median OS was 15 months. Three patients (14.29%) died because of tumor progression. The one‐year OS rate was 87.5%, and the two‐year OS rate was 76.6% (Fig 2a). Seven patients had progressive disease, and the median progression‐free survival (PFS) was 12 months. The one‐year PFS rate was 71.1%, and the two‐year PFS rate was 64.0% (Fig 2b). Three patients had local recurrence, and four patients had distant metastasis. The one‐ and two‐year local control (LC) rates were 92.9% and 92.9% (Fig 2c).

### Treatment‐related toxicity

**Figure 3:**
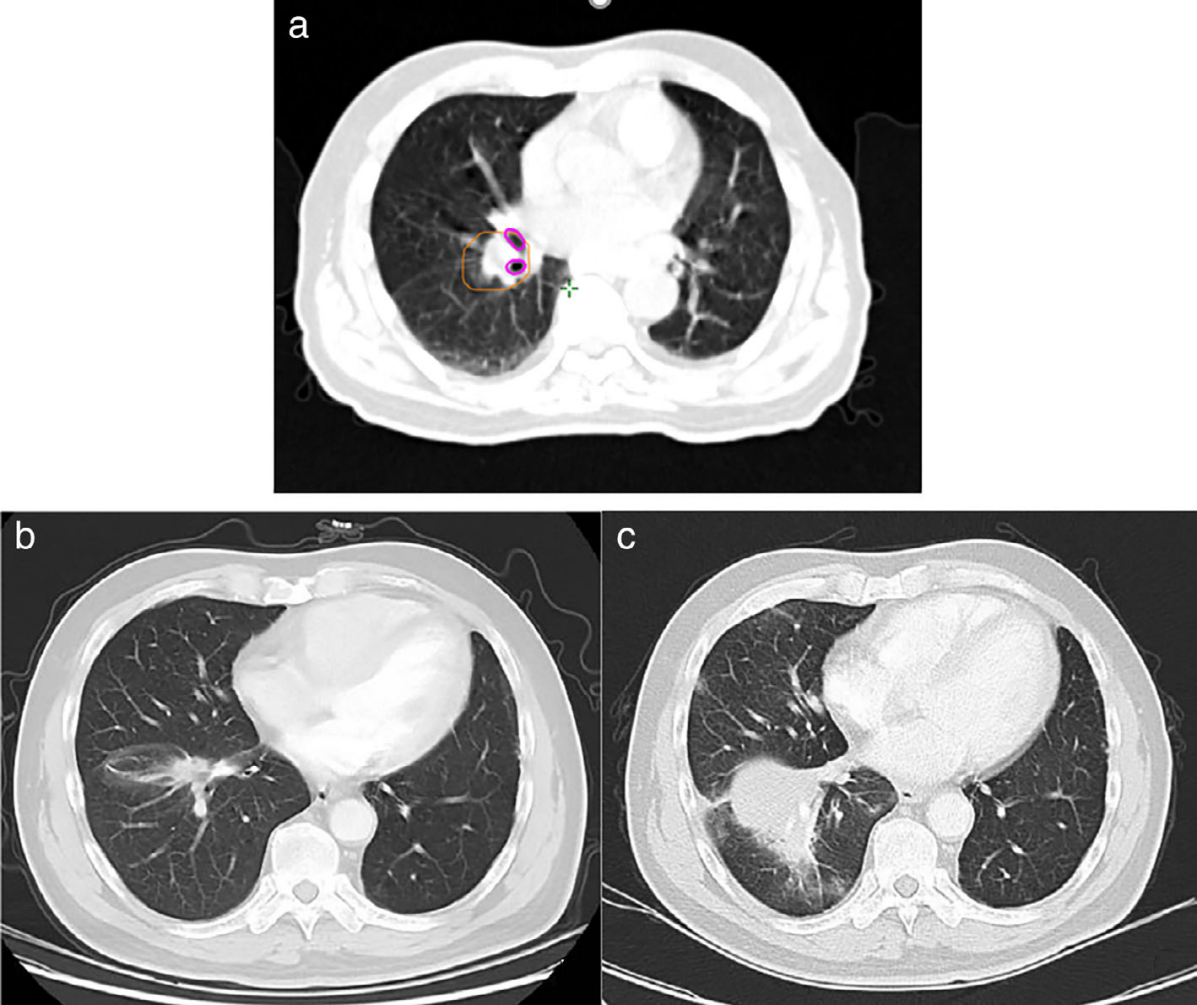
Radiotherapy plan and chest CT of the patient with grade 1 atelectasis. (**a**) Shows the radiotherapy plan (planning target volume in orange, right middle lobar bronchus and right inferior lobar bronchus in purple). (**b**) Chest CT before SBRT. (**c**) Chest CT after SBRT, which is in the same slice as (**b**) and shows grade 1 atelectasis.

The adverse events of radiotherapy treatment are listed in Table 2. A patient underwent chest CT four months after radiotherapy. The scan revealed enlarged right lower lobe nodules with malignant tendency and distal atelectasis (Fig 3). CT‐guided puncture showed granulomatous inflammation with massive necrosis, no clear tumor, and negative acid‐fast staining results. The patient was considered to have grade 1 atelectasis. Four patients experienced grade 2 radiation pneumonia, and 10 patients had grade 1 radiation pneumonia. The dosimetric details of bronchi for these 14 patients are shown in Table 3. One patient experienced grade 2 radiation esophagitis with dosimetric details that the D_max_, D_1cc_, D_2cc_ and D_5cc_ of esophagus were 19.19 Gy, 16.56 Gy, 15.30 Gy and 11.74 Gy separately. Two patients experienced grade 1 radiation skin injury. One patient had grade 2 myelosuppression. No patients experienced hemoptysis, hemorrhage, spinal cord injury or brachial plexopathy. No patients had grade ≥ 3 toxicities. All patients had grade 2 treatment‐related toxicities which occurred at a rate of 19.05%.

**Table 2:** Adverse events of radiotherapy

CTCAE v4.03	Adverse event	No. of patients (%)
Grade ≥ 3	All	0 (0%)
Grade 2	Radiation pneumonitis	4 (19.05%)
	Radiation esophagitis	1 (4.76%)
	Myelosuppression	1 (4.76%)
Grade 1	Radiation pneumonitis	10 (47.62%)
	Skin injury	2 (9.52%)
	Nausea	3 (14.29%)
	Fatigue	3 (14.29%)
	Atelectasis	1 (4.76%)

**Table 3:** Dosimetric details of bronchi for patients with radiation pneumonia

Grade of pneumonia	D_max_ (Gy)	D_1cc_ (Gy)	D_2cc_ (Gy)	D_5cc_ (Gy)
2	53.05	28.24	25.25	21.19
2	19.09	15.42	14.31	11.44
2	55.06	29.63	23.97	7.52
2	63.77	56.02	44.51	4.58
1	63.42	13.28	12.46	11.35
1	18.58	15.21	14.32	12.80
1	40.57	32.86	30.59	26.75
1	65.02	53.77	41.62	31.86
1	36.30	24.13	21.40	15.04
1	52.98	39.94	31.58	10.35
1	63.94	34.20	9.77	1.97
1	60.80	34.71	28.99	18.79
1	69.43	59.33	49.15	26.15
1	27.25	20.85	19.02	16.18

### Dosimetric analysis

**Figure 4:**
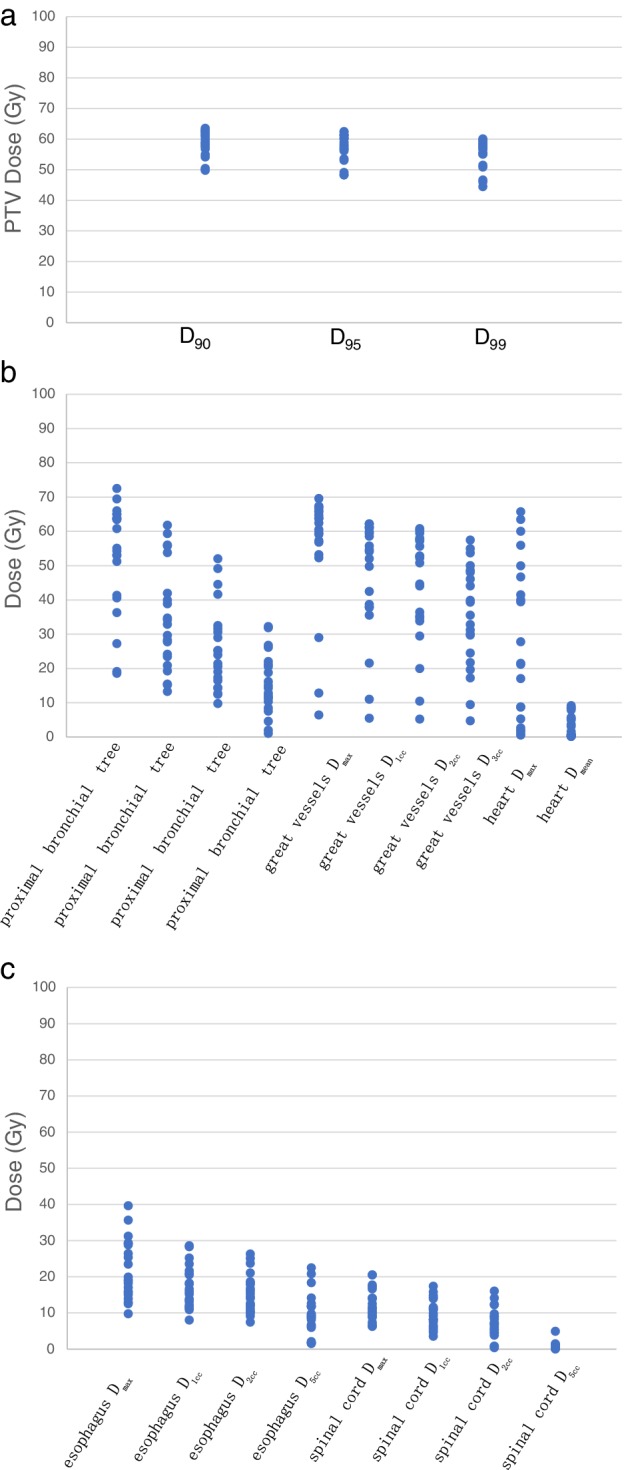
Physical doses for all patients.

Dosimetric details are summarized in Table 4 and Fig 4. The median PTV point D_max_ was 67.15 Gy (62.05–83.97 Gy). The median PTV D_90_ and D_95_ were 59.11 Gy (49.79–63.52 Gy) and 58.37 Gy (48.28–62.45 Gy), respectively. The median D_max_, D_1cc_, D_2cc_ and D_5cc_ of the proximal bronchial tree were 55.06 Gy (18.58–72.51 Gy), 32.86 Gy (13.28–61.78 Gy), 23.97 Gy (9.77–52.01 Gy), and 14.28 Gy (1.05–32.23 Gy). The median D_max_ of the great vessels and D_mean_ of the heart were 62.43 Gy (6.41–69.57 Gy) and 0.81 Gy (0.18–9.15 Gy). The median total lung V5 and V20 were 18.8% (6.78%–40.93%) and 7.88% (1.65%–17.99%), respectively.

**Table 4:** Dosimetric details

Dosimetric parameter	Median (range), Gy
PTV D_max_	67.15 (62.05–83.97)
PTV D_90_	59.11 (49.79–63.52)
PTV D_95_	58.37 (48.28–62.45)
PTV D_99_	56.95 (44.46–60.06)
Proximal bronchial tree D_max_	55.06 (18.58–72.51)
Proximal bronchial tree D_1cc_	32.86 (13.28–61.78)
Proximal bronchial tree D_2cc_	23.97 (9.77–52.01)
Proximal bronchial tree D_5cc_	14.28 (1.05–32.23)
Great vessels D_max_	62.43 (6.41–69.57)
Great vessels D_1cc_	54.12 (5.48–62.19)
Great vessels D_2cc_	50.78 (5.23–60.80)
Great vessels D_5cc_	35.54 (4.73–57.46)
Heart D_mean_	0.81 (0.18–9.15)
Mean lung dose	5.56 (1.64–9.89)

## Discussion

This study indicated that 60 Gy SABR in eight fractions may be safe for the treatment of ultracentral lung cancer. The rate of grade 2 treatment‐related toxicities was 19.05%. There was no grade ≥ 3 radiotherapy‐related adverse effect in the 21 ultracentral NSCLC patients treated in our center. One patient had grade 1 atelectasis, PTV overlapping the proximal bronchial tree, and PTV D_max_, D_90_, and D_95_ were 73.51 Gy, 63.52 Gy, and 62.45 Gy. The D_max_, D_1cc_, D_2cc_ and D_5cc_ of the proximal bronchial tree were 72.51 Gy, 61.78 Gy, 12.64 Gy, and 1.05 Gy, respectively. According to Table 3 and Fig 4, the proximal bronchial tree D_max_ of most patients was less than 70 Gy, and the D_1cc_, D_2cc_ and D_5cc_ were less than 50 Gy, 40 Gy and 30 Gy, respectively. The median D_max_ of the great vessels and D_mean_ of the heart were 62.43 Gy (6.41–69.57 Gy) and 0.81 Gy (0.18–9.15 Gy). There were no adverse events such as hemorrhage or heart‐related diseases. We conclude that these radiotherapy doses are safe for ultracentral lung cancer patients.

SABR is an effective treatment for patients with severe complications or pulmonary function who are unable to tolerate surgery. Using 4D‐CT and daily CT simulation to confirm the position and reduce deviation, SABR can deliver a high biological effective dose (BED) to the target while minimizing toxicity to the surrounding normal tissue, which may translate into improved LC and survival rate.^10^, ^16^, ^17^ With the progress of radiotherapy technology, SABR can be used to treat central lung cancer and even ultracentral lung cancer, which used to be risky to treat.

It has been previously determined that BED ≥ 100 Gy is required to achieve accepted tumor control.^18^ In our study, we delivered 60 Gy in eight fractions to the PTV, with a BED = 105 Gy (α/β = 10.0). In some radiotherapy plans, PTV dose was reduced because the PTV tightly overlapped with central structures. Overall, the dose of PTV was satisfactory, and the dose of OAR was within the normal tolerance range. The one‐ and two‐year OS rates were 87.5% and 76.6%, respectively. The one‐ and two‐year PFS rates were 71.1% and 64.0%, respectively. The one‐ and two‐year local control (LC) rates were 92.9% and 92.9%, respectively.

The optimal SBRT treatment for ultracentral lung cancer remains controversial. Tekatli *et al*.^12^ reported that it is safe and practical to treat single primary or recurrent ultracentral NSCLC with 12 fractions of 5 Gy. Median OS was 15.9 months, and three‐year survival was 20.1%. Grade 3 or higher toxicity was recorded in 38% of those patients. Raman *et al*.^14^ provided a regimen of 60Gy in eight fractions to a majority of patients (76.9%) with ultracentral lung cancer. The median OS was 23.8 months, and two‐year local failure rate was 0%. The rate of grade 2 or 3 toxicity was 7.9% and no grade 4 or 5 toxicities were observed. Murrell *et al*.^19^ suggested that 60 Gy in eight fractions was practical. Table 5 summarizes several other studies on SBRT for ultracentral lung cancer.

**Table 5:** Studies on SBRT for ultracentral lung cancer

Study	Regimen	Result	Toxicity	Remark
Timmerman *et al*.^20^ (*n* = 70)	20–22 Gy × 3	Median OS: 32.6 months Two‐year OS: 54.7%	Grade 3–5: 20% One patient died of massive hemoptysis	Include both peripheral and central tumors
Song *et al*.^21^ (*n* = 32)	10–20 Gy × 3–4 consecutive days, total of 40–60 Gy	One‐year OS: 70.9% Two‐year OS: 38.5% One‐year LC: 85.3% Two‐year LC: 85.3%	≥Grade 3 pulmonary toxicity in 33% of patients with central tumors Bronchial strictures observed in eight patients	Central versus peripheral
Chang *et al*.^22^ (*n* = 100)	12.5 Gy × 4 7 Gy × 10	• Median OS: 55.6 months • Three‐year OS: 70.5% • Three‐year LC: 96.5%	≥Grade 2 radiation pneumonitis: 13.4%, 5.5% ≥Grade 1 chest wall pain: 32%, 28% Brachial plexopathy: 3%, 0% ≥Grade 2 esophagitis: 3%, 0%	Two regimens: 7 Gy × 10 was used if constrains were not met
Tekatli *et al*.^23^ (*n* = 78)	7.5 Gy × 8	Median follow‐up: 47 months Three‐year survival: 53%	Grade 3: 6.4% Grade 4: 0% Treatment‐related death: 7.5%	—
Daly *et al*.^24^ (*n* = 46)	Range 40–60 Gy/4–8 fractions, median 10 Gy × 5	Median follow‐up: 21.4 months	≥Grade 3 toxicity in ultracentral tumors: 22.2%	In ultracentral tumors: • Two cases of grade 3 post obstructive pneumonia • One case of grade 1 respiratory failure

Compared with previous studies, the safety and efficacy of our treatment for ultracentral lung cancer are acceptable. However, there are some limitations to this study as it was retrospective with a small number of cases. Further clinical trials could be carried out to obtain more accurate clinical data. There are some patients who were followed‐up for a short time, and further follow‐up would be required.

## Disclosure

No authors report any conflict of interest.
